# Sexual Healthcare Preferences among Gay and Bisexual Men: A Qualitative Study in San Francisco, California

**DOI:** 10.1371/journal.pone.0071546

**Published:** 2013-08-19

**Authors:** Kimberly A. Koester, Shane P. Collins, Shannon M. Fuller, Gabriel R. Galindo, Steven Gibson, Wayne T. Steward

**Affiliations:** 1 Center for AIDS Prevention Studies, University of California, San Francisco, San Francisco, California, United States of America; 2 San Francisco AIDS Foundation, San Francisco, California, United States of America; Rollins School of Public Health, Emory University, United States of America

## Abstract

**Background:**

Research on gay and other men who have sex with men's (G/MSM) preferences for sexual healthcare services focuses largely on HIV testing and to some extent on sexually transmitted infections (STI). This research illustrates the frequency and location of where G/MSM interface with the healthcare system, but it does not speak to why men seek care in those locations. As HIV and STI prevention strategies evolve, evidence about G/MSM's motivations and decision-making can inform future plans to optimize models of HIV/STI prevention and primary care.

**Methods:**

We conducted a phenomenological study of gay men's sexual health seeking experiences, which included 32 in-depth interviews with gay and bisexual men. Interviews were transcribed verbatim and entered into Atlas.ti. We conducted a Framework Analysis.

**Findings:**

We identified a continuum of sexual healthcare seeking practices and their associated drivers. Men differed in their preferences for separating sexual healthcare from other forms of healthcare (“fragmentation”) versus combining all care into one location (“consolidation”). Fragmentation drivers included: fear of being monitored by insurance companies, a desire to seek non-judgmental providers with expertise in sexual health, a desire for rapid HIV testing, perceiving sexual health services as more convenient than primary care services, and a lack of healthcare coverage. Consolidation drivers included: a comfortable and trusting relationship with a provider, a desire for one provider to oversee overall health and those with access to public or private health insurance.

**Conclusions:**

Men in this study were likely to separate sexual healthcare from primary care. Based on this finding, we recommend placing new combination HIV/STI prevention interventions within sexual health clinics. Furthermore, given the evolution of the financing and delivery of healthcare services and in HIV prevention, policymakers and clinicians should consider including more primary care services within sexual healthcare settings.

## Introduction

*“I just want warmth, compassion, listening. I need that in a healthcare person.”*

Gay men and other men who have sex with men (G/MSM) face profound sexual health disparities, particularly in their increased risk of HIV and sexually transmitted infections (STI) [1], [2]. They accounted for 61% of all new HIV infections in the United States in 2010 [3], making them over 40 times more likely than other men to be HIV-positive [4]. Similarly, syphilis rates are estimated to be 46 times higher in G/MSM than in other men [4], while G/MSM constitute 10% of all new Hepatitis A cases and 20% of new Hepatitis B cases [5]. The Centers for Disease Control and Prevention recommends annual HIV and STI screening for G/MSM [6] and more frequent screening (every 3–6 months) for G/MSM with greater potential exposure to HIV/STIs. However, studies indicate that recommendations for HIV and STI testing are not being met [7], [8].

Addressing these critical health challenges requires a multi-component response that encompasses sexual health promotion, disease prevention, medical treatment, support for mental health and drug use challenges, and strategies to cope with a social context that has traditionally stigmatized sex between men [2]. Unfortunately, the current structure of the US healthcare system does little to promote the kind of integrated, comprehensive services necessary to address health promotion and disease prevention. One need only sample the patchwork of agencies serving G/MSM to see evidence of this challenge. Traditional HIV behavioral prevention, at least when targeted to HIV-negative individuals, has been centered in community-based organizations that offer few medical services [9]. Testing and treatment of sexually transmitted infections are frequently conducted in standalone STI clinics, due in part to historical and existing stigma against venereal disease [10]. HIV treatment is often provided in special healthcare delivery systems built up through the Ryan White HIV/AIDS Program [11]. While the Ryan White program ensures high-quality care [12], by statute it is available only to people living with HIV and thus effectively segregates HIV-positive G/MSM from HIV-negative G/MSM in care settings. Mental healthcare and drug abuse services are spread across agencies, clinics, and private practitioners. Such services may be constrained by insurance coverage gaps, inadequate infrastructure, and stigma against mental illness and drug use [13], [14]. Finally, programs intended to mitigate the impact of prejudice or address other structural challenges (e.g., absence of housing) are often placed outside of the healthcare system altogether, being the province of political advocacy groups or of legal aid organizations. Against this backdrop, G/MSM, as well as all other persons facing similar circumstances, must find their way to the services that they need.

Research to date on G/MSM and sexual health services has primarily focused on HIV testing [15], [16]. This research provides a snapshot of where G/MSM interface with the healthcare system to address HIV risk and other sexual health concerns. But they do not speak to why men sought care in specific locations or to how that care might fit within broader (non-sexual) healthcare needs. The UNAIDS campaign *“Know your epidemic, Know your response”* [17] remains highly relevant when considering how to compassionately and effectively respond to the continuing HIV epidemic. In addition to knowing the epidemiological risk factors, an equally important aspect of “knowing your epidemic” is, in this case, to understanding *how* G/MSM manage their sexual health within a healthcare environment such as the one described above. This type of research provides findings that are essential to informing the design and implementation of newly emerging HIV and STI combination prevention strategies. Combination prevention approaches are already widely used for controlling infectious diseases such as tuberculosis and malaria. Recently this concept has been tested for use in HIV prevention [18]. Research is underway to identify which combination of elements produces successful outcomes [19], [20], [21], [22]. Combinations will certainly differ and depend on geographic location, target population, cultural factors, and available resources, among other things. In the US, combination approaches will also cut across traditional service delivery domains, and require enhanced coordination among providers and/or the development of new capacities within existing agencies. As an example, consider comprehensive HIV testing, linkage to care, plus treatment (TLC+), otherwise known as “treatment as prevention” [23]. This strategy may successfully prevent HIV by placing an infected person on treatment to lower viral load and thereby reduce infectivity [24], [25]. But it only works if an infected individual is first tested and diagnosed, successfully linked to care, retained in care, prescribed antiretroviral medications, and then appropriately adheres to the prescribed regimens [26]. Substantial loss-to-follow-up occurs at each of these steps along the treatment continuum [27]. TLC+ thus requires coordination among testing and treatment sites to ensure successful linkage to care. Furthermore, to the degree that a person's engagement in care and/or adherence to medications is hindered by mental illness [28], drugs [29], [30], and/or structural challenges such as, absence of housing [31], TLC+ may also require coordination with case managers, mental health professionals, and agencies that address structural barriers.

As a second example, consider pre-exposure prophylaxis (PrEP). It involves the administration of an anti-retroviral (ARV) agent to HIV-negative individuals to prevent acquisition of the virus [32], [33]. Traditionally, ARVs have been prescribed to HIV-infected individuals and monitored within doctor's offices and clinics. But many HIV-negative G/MSM do not have ongoing ailments and, thus, may not regularly see a medical provider or otherwise seek medical care. If and when HIV-negative G/MSM do seek testing and other services, they may do so at STI clinics or community agencies that offer preventive services to uninfected men but that are not typically equipped to provide continuity of care or to prescribe medications. Furthermore, because PrEP does not protect against other sexually transmitted infections, it may be ideally combined with other services (e.g., behavioral counseling for risk reduction), thus necessitating that delivery sites have expertise in both biomedical and behavioral intervention strategies [34]–[36]. Infrastructure to accommodate the particularities of delivering PrEP is underway; it is unclear how much effort is necessary to integrate PrEP into current healthcare delivery systems. Once built, it remains to be seen to what extent G/MSM will avail themselves of these services.

As HIV prevention moves toward more integrated models, understanding G/MSM's experiences, motivations and decision-making around receipt of sexual healthcare services can inform where best to situate new services (e.g., delivery of PrEP) and how best to coordinate services across agencies. In this phenomenological study [37], we sought to explore gay and bisexual men's sexual health seeking experiences, how they decided where to obtain HIV testing and other sexual health services.

## Methods

We conducted in-depth interviews with gay and bisexual men in San Francisco between January and June, 2010, as part of a larger mixed methods study to evaluate the community level impact of a sexual health center (Magnet) devoted to promoting the physical, mental and social well-being of gay men. The center is located in San Francisco, California, which has a population of approximately 800,000 residents and is well known for its vibrant lesbian, gay, bisexual, and transgender communities. The goals of the analyses presented here were to identify the range of resources that men used for the purposes of sexual health promotion and then to describe and characterize men's sexual health seeking experiences. In other words, we wanted to understand where men were going for sexual healthcare and why.

### Recruitment

To advertise the study, we used fliers placed in venues frequented by gay men, online advertisements, and word of mouth recommendations from Community Advisory Board members affiliated with our research institution. Men interested in participating called a toll free phone line to discuss with a researcher whether they were eligible for the study. Importantly, enrollment in the interviews was not dependent on use of Magnet services. Rather, eligibility criteria were designed to capture the broader local community to which Magnet potentially offers services. Specifically, participants had to: be English-speaking; be 18 years of age or older; self-identify as male; self-identify as gay or bisexual; and report having sought out sexual health services in the San Francisco Bay area. Participants were also required to live, work and/or to socialize at least weekly in San Francisco. The study was open to men of both negative and positive HIV serostatus. Both groups of men receive sexual health services at Magnet and we wanted to capture the experiences of all gay and bisexual men regardless of their HIV status. The overall study focused on the evaluation of a sexual health services clinic and community center for gay men, therefore recruiting gay men and other men who have sex with men was warranted. There were no inclusion/exclusion criteria based on race or ethnicity.

### Research team

Qualitative interviewing and analyses were led by a heterosexual female, cultural medical anthropologist with over ten years of experience conducting ethnographic and qualitative research on HIV prevention particularly with gay men and HIV care specialists. Assisting in the recruitment, interviewing and initial analysis was a gay male, doctor of public health with a decade of experience working on health disparities research projects among ethnoracial and sexual minority communities. Each participant was screened by one of the two researchers. Notes about each caller were captured in an Excel file (e.g., source of referral, whether the caller sounded able to give consent). These initial screening conversations allowed us to assess the level of interest in the subject matter as well as to assess whether the caller was talkative and coherent (in one case, the caller sounded to be high on stimulants and was overly talkative and incoherent at times).

### Interview Process

We scheduled participants for an interview at a mutually agreeable time in a private office located in our research center. Prior to initiating the interview, we provided participants with an information sheet and asked them to provide verbal consent which was documented with the interviewer's signature. All study procedures were reviewed and approved of by the Committee on Human Research at the University of California, San Francisco. This committee allowed us to gather verbal consent due to the privacy risks associated with the study. In addition, the information sheet stated that access to the transcribed interviews would be limited to the members of the immediate research team, a condition we imposed because the interviews contained information about specific events and services. Given the extent of detail provided in the narratives, it would be difficult for us to guarantee full anonymity if the complete transcripts were made publicly available. Interviews were conducted face to face with a single interviewer and the participant. To optimize comfort, we gave them the option of being interviewed by either the female or male interviewer (KK or GG). We drafted an open-ended interview guide and pilot tested it with two men. We revised the guide and made spontaneous modifications to it when appropriate e.g., dropping questions that were not applicable. We asked participants to describe their history with sexual health seeking experiences and then to focus on describing in-depth at least one recent experience. Interviews lasted between 45 to 90 minutes and were subsequently transcribed verbatim and checked for accuracy.

### Analyses

We conducted a Framework Analysis [38] which includes a multi-step process of reading and re-reading the data, applying a coding scheme which consisted of both inductive and a priori codes, code interpretation, theme identification, generating tables in order to compare narratives and returning to a subset of interviews to be read in full to maintain analytic holism. All interviews were entered into Atlas.ti [39] to expedite the process of sorting and organizing the data in preparation for coding and theme identification. Authors KK and GG conducted the initial coding, code interpretation and theme identification during analysis meetings held over the course of 4 months. Later, SC and SF participated in the refining and validating of the themes by reading and re-reading full transcripts. We generated a total of 27 codes. For this analysis, we present the themes associated with the following codes: sexual health seeking narrative, decision-making, and primary healthcare narrative. Once we constructed our themes, we conducted a members-check to ensure our interpretation of the data were accurate [40]. The members-check included a review of the findings with two key informants as well as a presentation to a group of Magnet volunteers.

To ensure rigorous application of qualitative methods, analysis and presentation of study findings, we followed the COREQ checklist [41].

### Findings

In total, 64 men called to be screened. We made three attempts to return the phone call of each person who contacted us. We ultimately screened 41 individuals, 9 of whom were deemed unsuitable as informants (e.g., high during screening call, hostile during the call). Of these 32 enrolled participants, three were female-to-male transgender individuals who identified as MSM. Due to their unique healthcare needs, we analyzed these data and plan to publish these data separately. The remaining 29 participants ranged in age from 20–67, ten self-reported as HIV positive, 18 self-reported as HIV negative, and one self-reported his HIV status as unknown. Table 1 provides a brief description of the participants quoted below. All names are pseudonyms.

**Table 1:** Information about Quoted Participants and the Interviewers.

Participant ID	Ethnicity	HIV Status	Typology	Interviewer
Luke	Native American	HIV-negative	Fragments	Male
Keith	African American	HIV-negative	Fragments	Male
Brad	White	HIV-negative	Single-Issue, insured	Female
Kurt	White	HIV-negative	Single-issue, uninsured	Male
Daniel	White	HIV-negative	Opportunist	Male
David	Latino	HIV-negative	Opportunist	Male
Greg	Bi-racial	HIV-negative	Consolidates	Male
Craig	White	HIV-negative	Fragments	Male
Anthony	Latino	HIV-negative	Opportunist	Female
Brian	White	HIV-negative	Fragments	Male
Alex	Latino	HIV-negative	Opportunist	Female
William	White	HIV-positive	Fragments	Female
Luis	Latino	HIV-negative	Opportunist	Female
Paul	White	HIV-negative	Fragments	Female

Note. All Participant IDs are pseudonyms.

### Continuum of Sexual Healthcare Seeking Practices

**Figure 1:**
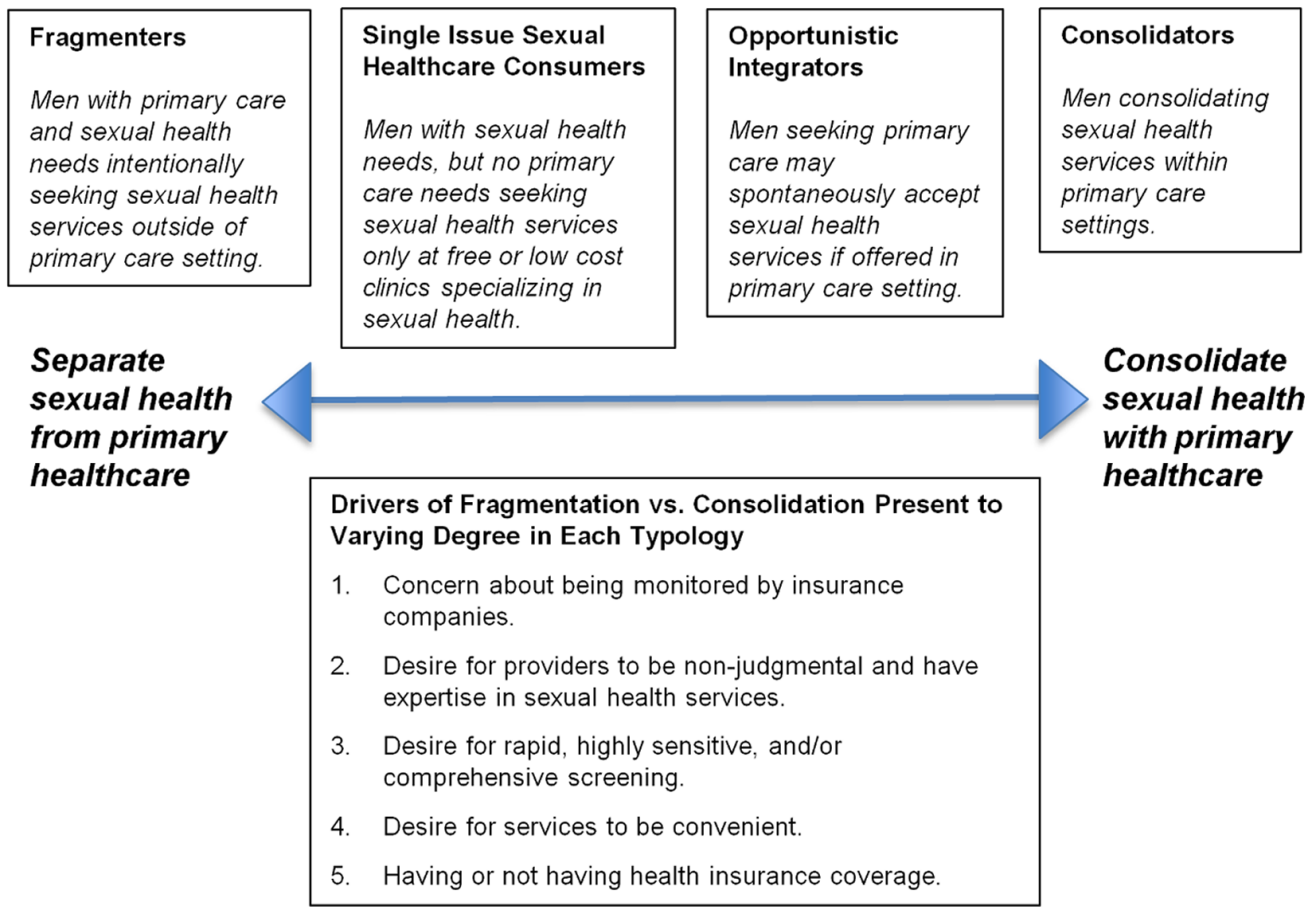
Continuum of Sexual Health Care Seeking Behaviors/Typologies Among Gay Men.

We identified a continuum of sexual healthcare seeking practices and their associated drivers. In our sample, men differed in their preferences for separating sexual healthcare from other forms of healthcare (a practice we labeled “fragmentation”) versus combining all care into one location (a practice we labeled “consolidation”). These behaviors effectively divided them into four typologies (behavioral profiles), based on the degree to which they fragmented or consolidated care. The typologies include: fragmenters, single-issue sexual healthcare consumers, opportunistic integrators, and consolidators. A definition for each typology is provided in Figure 1. The figure also lists the key factors that drive men toward fragmentation or consolidation.

#### Fragmenters

At one end of the sexual healthcare seeking continuum, we placed men who intentionally fragmented their care and emphasized separation of sexual health services from primary care. Most participants in the category were HIV-negative. Insurance status was mixed, including both uninsured and insured men. We identified several overarching factors that drove men to seek testing for STI and/or HIV in locations separate from their primary care provider. These included a desire to test for HIV in a setting that offered rapid HIV testing technologies, concerns about insurance companies monitoring behaviors associated with HIV and STI testing or diagnoses, a preference for talking to a professional sexual health expert, convenience and, finally, a lack of health insurance coverage. Sometimes men opted to fragment their care because of concerns about how their primary care provider would react to whatever sexual health concern or issue they were facing. Rather than enduring possible embarrassment or loss of respect, men would turn to sexual health clinics as alternative options. Below we provide two case illustrations of attitudes and behaviors we commonly observed among our “fragmenters.” First, we asked “Luke” whether he had a reason why he attended a sexual health clinic for HIV testing and/or STI testing as opposed to going to his primary care physician, a line of questioning pursued during interviews with men who recounted stories of fragmenting their care. He stated:

It sounds funny, but you know, it's the whole social stigma of not having my primary –I cared about what my primary care physician thought about me, which is really stupid now I realize. Like, oh, shame and everything. But you know, just the people in the office kind of became friends. They'd look and, “Oh, there's Luke. We're so happy that Luke's coming in today.” And then, oh, yeah, I'm here for, you know, gonorrhea. It was just like I didn't feel comfortable with that.

In this case, the participant was reluctant to face the potential for spoiling his identity [42] among the clinic staff by requesting treatment or testing for an STI, something he perceived as stigmatizing.

In the case of Keith, when asked a hypothetical question about whether he would feel comfortable going to his primary care provider for an STI test if the local sexual health clinic was closed, he stated:

I'd probably feel less comfortable. I guess I feel like when you go into a sexual health clinic, the idea that you're there for a reason has already sorted out. Whereas, if I were to go to my doctor and sort of approach him about a sexual health concern, I feel like I'd be a little more uncomfortable about it. Just not wanting to reach that personal level with him, or something. I don't know. I guess he like, sees my body and stuff. So, it doesn't really make any sense.

In the cases above, both men fragmented their care, but were self-effacing about the behavior during the interview claiming that it was “stupid” or “doesn't really make sense.” This may indicate that in these two particular cases, when given the opportunity to reflect on their choices, they either downplayed or were unable to articulate feelings of vulnerability associated with being gay and with feeling uncomfortable raising sexual health issues in a primary care setting. Instead the narrative used to account for fragmenting sexual health and primary care was to assign self-blame.

#### Single-Issue Sexual Healthcare Consumers

Next along the continuum, we identified a group of HIV-negative men who entered into healthcare for only one reason – to seek out sexual health services. We labeled this health-seeking typology as: single-issue sexual healthcare consumers. Men in this category were exclusively HIV-negative. They were also relatively more likely, but not exclusively, uninsured. However, regardless of insurance status the men's healthcare seeking pattern appeared to be driven by a perception that their healthcare needs were limited to sexual health concerns and by the ready availability of such services in community-based settings, typically for free. Through these choices, the men were setting up a pattern of care seeking that would effectively fragment their care were they to also need non-sexual health services (because those services would have to be obtained in other settings). Conceptually, men in this typology category could be distinguished from fragmenters by their motivations. Unlike fragmenters, the observed pattern of single-issue sexual healthcare consumers was *not* centered on a desire to separate sexual and non-sexual health services. Rather, they described a singular healthcare need and explained that, by and large, this need could be managed in one location – a community-based clinic offering HIV/STI testing and treatment. In the first illustration of this type, Brad is a 26-year-old participant with health insurance; when asked about his use of primary healthcare he stated:

I still haven't gone – my work provides me with Anthem. . . . I have that insurance and that option, but most of my issues are sexually related and luckily, there are free clinics for that.

Kurt did not have health insurance at the time of our interview and although he had had a private physician in the past, for the sake of ease and economy, he always turned to public health services for STI screening and treatment and HIV testing. When asked whether he had ever had STI screenings with his private physician he responded:

No, there was no need. It was easier and more – economy to the health service. There's no charge for the services, so that's always a plus when you're uninsured. And it was just always easier to get an appointment, and that's also a plus, which isn't necessarily always true with a physician. That's primarily why. The services were good; they were efficient.

#### Opportunistic Integrators

Our third typology included a subset of men that had established relationships with primary care providers (PCPs). The participants in this category were relatively more likely to be insured and were exclusively HIV-negative. Like men in previously presented typology categories, they actively planned to test for HIV or STIs on an as-needed or routine basis in community-based testing sites unaffiliated with primary care practices. They also visited their PCPs for various non-sexual health concerns or screenings (e.g., an annual check-up or for a medical complaint). What distinguished the men in this category was their acceptance of sexual health screening if it were offered to them during a primary care visit. We labeled the group “opportunistic integrators” to reflect their willingness to take advantage of the primary care visit as an opportunity for sexual healthcare, but also to acknowledge that the integration of sexual and non-sexual healthcare needs was not a result of their own intentional planning. For example in the case of Daniel, his sexual health maintenance consisted of testing in two free clinics catering to gay men. He explained the occasions when he was willing to break out of that routine:

A couple of times I've gotten HIV tests via my primary care physician, just because I was there and I was getting blood drawn, just more for convenience. I don't recall ever seeking treatment for an STD, or what I perceived to be an STD via my primary care physician.

Another participant, David, illustrated well the opportunistic nature of testing during a PCP clinical encounter by describing a theoretical situation in which he might be willing to test for HIV:

For instance, if I had my doctor here in the city and I need to get tested, I'm still going to go to Magnet. I'm not going to go to my doctor unless we're in the middle of something like that and then he says at some point, I think you should get tested. And I think I would be okay with that.

#### Consolidators

The final typology comprises men who described a pattern of actively planning to conduct all sexual health services in the context of primary care. In our sample we encountered very few cases of HIV-negative men who fit this typology. The majority of “consolidators” were men living with HIV for whom consolidation of STI screening, diagnosis and treatment was ostensibly easy to achieve within the context of routine HIV care, assuming they were in care and their provider was following the HIV Medicine Association's primary care guidelines [43]. We noted that among HIV-negative men, those who were insured and described having a trusting relationship with a healthcare provider seemed more inclined to consolidate sexual health within the context of primary care. One HIV-negative man who had a background in healthcare work, Greg, explained why he preferred to go to his primary care provider for his sexual health needs:

Just because I feel like he's the person I'm trying to organize my health, my overall health history with, and I want to keep him in the loop with everything. And he's gay. I don't feel like I can have a whole lot of explaining to do or anything like that with him.

It should be noted that there was no observed relationship between insurance status and being a consolidator. This appeared to be due to HIV-positive men having access to primary healthcare, regardless of whether or not they were insured. Even men who were uninsured reported some form of healthcare coverage, usually through publicly funded mechanisms like the Ryan White HIV/AIDS Program.

### Exploring the drivers underlying sexual health seeking practices

We identified five drivers that shaped the way participants went about seeking sexual health services. These drivers are intertwined with one another and are not necessarily associated with one typology or another. The majority of our participants invoked one or more of these drivers while being interviewed.

#### 1. Fear of being monitored by insurance companies, employers or the government

*“I don't want my HIV test results in my medical records.”*

Craig dramatically compared his preference for fragmenting sexual health and primary care to “separat[ing] church from state.” His concern was that insurance companies would discover that he had a history of testing for HIV and would then prejudicially refuse to offer him insurance coverage. We noted this sentiment over and over again in the interviews with men who fragmented sexual healthcare from primary care.

For instance, Anthony, who had health insurance and sought out gay-friendly providers, expressed a desire to test in the context of his primary care provider, but explained that his concerns about retaining his insurance coverage prevented him from doing so. Like Craig, he feared loss of insurance or becoming uninsurable in the future. He also indicated that he perceived this to be normative behavior. His case below highlights this pervasive concern, which frequently drove men to test in settings without ties to one's personal health record:

Interviewer: Tell me about why you don't get an HIV test with your doctor.

Participant: I was told that HIV tests – the insurance companies have access to those records and they can deny you if they see that you have HIV, when you're – if you're testing, that means you have some kind of risk for HIV, and they might deny coverage and it's in your records. They've never been clear. Some say yes, some say no. I remember one doctor told me not to do that. *A lot of people don't do tests with medical providers [emphasis added].*

#### 2. Desire for specialization in sexual health services

Some participants were very particular about selecting competent providers that they felt matched their own level of sophistication around sexual health and HIV prevention. Alternately, some participants chose sexual health clinics for their care or because they were mistreated in the past by homophobic or heterosexist providers and were actively seeking providers they could trust to be gay-friendly. Although several participants indicated that they had gay or gay-friendly PCPs, others noted that they had more extensive, more meaningful conversations about sexual health in public clinics focused on sexual health. Brian, who was an insured patient of a well-known gay PCP, yet still fragmented his sexual healthcare from primary care, stated:

I guess I was more embarrassed to go to my doctor about STDs, and Magnet was so open and accepting and loving, that you're just like, oh sure, everyone has gonorrhea, so come here. And so that felt better to go to Magnet. Just because it was a more accepting atmosphere…. I think it is the rapport, and they do listen pretty well. They hear what I'm saying. Again, they're not condescending. They match my level of literacy about sex – gay men's sexual health. So, like if they feel like I know – they don't give me a lecture. I think they can tell what I know as I'm talking, and so they don't repeat a lot of stuff I don't need to hear, or – they're just good at reading me. Like they're hearing and listening to me…. so sometimes I appreciate having a gay male person, because I think – [they] understand what I'm going through, what my life is like as a sexually active gay man. ...I just want warmth, compassion, listening. I need that in a healthcare person. …I don't want condescending …Because it's just a vulnerable situation and you need someone who's super sensitive, is my feeling.

Daniel, a participant in the “opportunistic integrator” typology, had health insurance and a relationship with a provider who primarily cares for gay men. However, he described his preference for going to a local gay men's sexual health clinic rather than his gay primary care provider:

It's not that I don't trust my primary care physician, but I feel like it's – I don't know. Especially since that I've been going to Magnet. I'm going to have a good conversation with someone else about sexual health practices and get free condoms. There's more value to me in going to Magnet than going to primary care physician if the issue is something related to an STD or sexual health because there's just more specialization of that. It seems like they might be more up on the latest, what's happening in San Francisco, and things to watch out for that.

Craig, Daniel, and several other participants also described instances in which they were offered and accepted preventative health services (i.e., hepatitis vaccinations) at a sexual health clinic:

I went and I got the series of Hepatitis shots at Magnet.

It didn't seem like I was super high risk, but it seemed like the side effects of the shots were pretty negligible and the harm they prevent against is pretty severe […] I just felt like it was probably the prudent thing to do, is get the shots. –Daniel.

I'm not really a fan of needles like I said, and, you know there's the [hepatitis] A and the B [vaccines], so you have to do two separate ones on two separate occasions. But, when I was talking to the nurse she said, “Well you know we have a combination.” I'm like, Sweet. And I'm like, All right, cool. –Craig.

These interactions illustrate how these men regarded their sexual health clinic as a trustworthy source of sexual health advice, and demonstrate that sexual health centers can reach those at risk for hepatitis A and B.

#### 3. Desire for rapid and/or highly sensitive and/or comprehensive screening technologies

Each participant described a few different experiences of HIV testing, including their first and most recent HIV tests. Men unanimously expressed a strong preference for rapid testing, and noted that the availability of rapid test technologies directly influenced their choice of testing venue. For example, Alex, an opportunistic integrator with health insurance, explained that, in general, primary care clinics do not offer rapid HIV testing while sexual health clinics do:

At (physician's office), they don't have the rapid testing – they have to draw blood. Sometimes when I didn't want to go in for a test, I'll just go into Magnet. I'll get it done in 15 minutes. It'll be a swab. It'll be rapid testing. I'll know, rather than going to (physician's office), making an appointment for next week – especially when you're experiencing a lot of anxiety or whatever – having blood drawn and then waiting two weeks to get the results and then having your doctor call you because they can't say anything in the mail or whatever so I was like, okay, I'll just go to Magnet.

Brian, a fragmenter introduced above, corroborated Alex's sentiment regarding the high level of anxiety caused by waiting to hear about an HIV test result:

I can't handle anything but a rapid test. Waiting a week is like, put me in a coma. I don't want to wait a week to hear. I'll die. That's awful. So, yeah, I have to have the rapid. And yeah, make an appointment? Magnet, you can go in, get it that same day. You don't have to wait two weeks to go into the doctor.

A few of our participants were accustomed to RNA HIV testing. RNA HIV tests are highly specific and can detect HIV prior to antibodies becoming present. Whereas a typical rapid HIV test detects antibodies at 4–6 weeks of exposure, RNA tests can detect HIV as early as 10 days following exposure [46]. While RNA HIV testing is typically expensive and not widely available, some men in our sample specifically sought it out in order to ameliorate their serostatus anxiety– a state of mind in which an individual experienced high levels of anxiety and heightened concern about their HIV status, typically following a sexual encounter they thought may possibly have exposed them to the virus. Men explained that the best way to combat the stress associated with these feelings was to submit to an RNA test for quick and definitive diagnosis, or to seek out post-exposure prophylaxis (PEP). One of our participants, Brad, a health insured, “single-issue sexual healthcare consumer”, described the benefits of RNA testing:

Interviewer: What's the benefit of doing RNA?

Participant: Not putting yourself through anxiety of three months for the window period, and knowing, I think, 10 to 14 days after exposure. I mean that test still takes like up to two weeks, so you still have to wait kind of like a month after exposure, but it's better than three months.

Some men also described a desire to seek care in a medical setting with on-site laboratory services because these conditions allowed for immediate and definitive diagnoses of STIs. These types of services, RNA testing (sent to an off-site lab with capabilities to screen for RNA) and/or on-site labs, often motivated men to select a particular location in which to seek care.

In addition, some men wanted a comprehensive exam including a rectal or throat swab rather than a urine test to ensure they did not have an STI. They explained that if this were not offered in a primary care setting, they would turn elsewhere to find these specific services:

When I would see my regular doctor on a regular basis, I would tell him I want to be screened for STDs to make sure I don't have anything. They didn't do any anal or oral swabs for a year. And he kept telling me that I didn't have any STDs. He'd say, “Oh, you're fine. No STDs. Because everything came out normal.” And then when this throat–when I went to Magnet, he says, “Oh, no. You have to do a swab on your anus and your throat to make sure you're completely clear.” And I go, “My doctor doesn't have me do that.” And he goes, “Well, then you're not necessarily STD free.” So that's how–I had a gonorrhea problem for a long time in my throat.” And I went back to my doctor and I was really upset. I said, “How come you never checked me?” He said, “We never–because you never told me to do that.” And I go, “Well, you're my doctor. You should be saying to me–you should be saying, Hey, we should do this as precautionary.” I mean, so I was a little taken aback by that. –William.

#### 4. Desire for services to be convenient

Convenience was a major determinant of where men sought sexual health services. However, it is important to note that “convenience” was differently defined for each individual. Whereas some men pointed to care locations in close proximity to where they spent time or to locations that had desirable hours of service, others highlighted the convenience of the services provided, such as shorter waiting times, the use of appointment vs. drop-in scheduling, and the existence of on vs. off site laboratory facilities.

Most men liked the proximity of a clinic embedded in the gay neighborhood, as explained by William, an opportunistic integrator:

Sometimes it's difficult to get in with my doctor. …I'd have to go there, get the lab slips, take them and then go to another company. Whereas Magnet is so easy and convenient; I got a number, I came in, I waited, and they just did it right there. And plus it's just down the hill, so you can have lunch and be with friends. It was a lot more convenient and I thought the people that work there actually are pretty good.

However, for some participants, convenience was also seen as a barrier. The prominent and easy-to-access location of Magnet provoked concerns about being seen in the clinic, and the potential stigma and loss of privacy that may result from this. Luis, an opportunistic integrator articulated this concern:

I don't want to go in there and be sitting down, waiting for my STD test to come back, and my boyfriend's best friend walks in, and goes, “ooooh shit,” you know what I mean? …I'd rather not know the person who I'm going to be working with. If I accidentally end up getting gonorrhea, I sure as hell don't want some gossipy queen knowing about it. I do not want that happening. I'd rather go to [the public STD clinic] and work through the machine where it's all anonymous, take my number, sit down, have someone I don't know.

#### 5. Lack of healthcare insurance coverage

Publically funded STI clinics are designed to serve individuals regardless of insurance status. A number of men we interviewed did not have health insurance and were more likely to receive sexual health services in a standalone STI clinic than free or affordable primary care (e.g., via publically funded primary care health centers). This made it more probable that they were found in the typology category of Single-Issue Sexual Healthcare Consumer and less likely to be found in the category Opportunistic Integrator (which, by definition, required one to be receiving care from a PCP).

Importantly, the influences of insurance status on typology category were seen only among HIV-negative men. HIV-positive men were much more likely than HIV-negative men to be consolidators of care and to choose this behavioral pattern regardless of insurance. That pattern appeared to be due to such men having guaranteed access to care through public funding sources, such as the Ryan White HIV/AIDS Program.

Insurance status was also uncorrelated with the fragmenter typology category. And reflected in the quotes below, participants from this group did not characterize their decisions as being due exclusively to insurance status. Furthermore, their behavioral patterns around sexual health services persisted even when uninsured men subsequently gained insurance coverage.

I didn't have insurance at the time and I also felt more comfortable going to a place that was specifically centered around sexual health, as opposed to seeing a primary care physician. – Keith.

When I discovered Magnet a couple years ago, I didn't have health insurance. Now that I have health insurance, I still go to Magnet, because A. I feel comfortable there and B. I believe in what they do and C. It's a safe environment. – Paul.

## Discussion

We identified a continuum of sexual healthcare seeking practices among gay men. Along the continuum we identified four typologies. The *fragmenters* intentionally maintained sexual health services separate from primary care services. The *single-issue sexual healthcare consumers* limited their care to sexual healthcare settings because they only utilized sexual health services. The *opportunistic integrators* were men with flexibility in where they obtained sexual health services. They loosely spoke of preferring sexual health services in standalone locations, but were responsive to offers of HIV/STI testing in primary care settings. Finally, the *consolidators* made a systematic choice to integrate sexual health and primary care.

We noted that among the different typologies, intention and behavior differed. Anchoring the continuum, we identified the fragmenters and consolidators, both of whom aligned their intentions and behaviors in that they intended to either separate or combine sexual health and primary care and then acted accordingly. In contrast, the single-issue sexual healthcare consumers and opportunistic integrators had less clear intentionality when seeking sexual health services. If single-issue sexual healthcare consumers were to seek other forms of care, that care would necessarily have to be fragmented from their sexual health services, if only because sexual health clinics generally do not offer other forms of care; however, the evidence for *intentionality* of fragmentation is less strong than for true fragmenters. For opportunistic integrators, intention and behavior did not always align. Their preferences are in part a reaction to the social environment, as they understand it. They see HIV testing as something a person does in a standalone clinic, not in the doctor's office, but they will take the opportunity to be tested if the primary care provider offers or recommends it and the conditions are right i.e., sufficient time, trust in provider.

The various typologies were influenced not only by intentions, but also by HIV serostatus as well as the structure of the health system. For example, most of the “consolidators” were men living with HIV. Given the design and organization of their healthcare system (i.e., Ryan White Program), it is reasonable for them to expect to have all services in one place and to actively reject a strictly fragmented care model. However, they do fragment care some of the time for some of the same reasons that HIV-negative men do – namely, out of convenience. By contrast, the healthcare system is not set up to easily consolidate services for HIV-negative men. Many participants did not think of their primary care provider as the logical source for HIV testing, even among those who had agreed to be tested in the context of a previous primary care visit. Other HIV-negative participants had limited access to primary care, owing to a lack of insurance. This made it more likely that they had sought out only sexual health services because such services are usually delivered for free in standalone STI clinics or community-based organizations.

In returning to the issue of combination HIV prevention strategies, these findings have implications for emerging prevention models, which require expertise that has traditionally been situated in varied environments (community-based agencies, HIV testing sites, doctors' offices). Young HIV-negative men have limited reasons to interact with the healthcare system, thus favoring models that focus on the delivery of discrete services (e.g., STD clinics, HIV test counseling) rather than on the establishment of enduring care relationships. Furthermore, doctors' offices are busy and their practices shaped by insurance considerations. Given these realities, there is little reason to expect HIV-negative men to place a premium on consolidation, especially when they are experiencing a healthcare system that does not actively promote such a concept.

Our findings speak to where men would be likely to access sexual health services. While we agree with others who have stated that, “Primary care clinicians are in a strategic position to provide HIV and STD screening and counseling services to MSM,” [44] our findings suggest that this may not be enough to ensure adequate access to services, particularly for men who are not infected with HIV. A primary care clinician may be in a “strategic position” to serve MSM, but there is no guarantee that an HIV-negative gay man will come to see that provider. Many of the men we interviewed resisted testing for HIV in primary care settings out of fear. They were reluctant to engage in meaningful conversations until they had established that a provider understood sexual health issues of gay men and could remain non-judgmental. Other men wanted reassurance that they would not be monitored and/or discriminated against by insurance companies or employers for seeking sexual health services in primary care settings (a concern that may lesson after implementation of healthcare reform). These findings suggest that combination prevention models may be better situated in standalone sexual health service centers that both HIV-negative and positive men are already using.

This research also has important implications for sexual health clinics offering interventions designed to reduce gay-related health disparities. Prior research has established significant medical and psychosocial disparities within the gay community. These disparities are varied. Heart disease, some cancers, substance misuse, smoking, depression, anxiety, eating disorders, intimate partner violence and suicidal ideation are among the illnesses gay men experience at higher rates than heterosexual men [45]–[71]. These disparities are a byproduct of stigma, discrimination and social marginalization [45]. Sexual health clinics, as environments in which gay men seek care, may be well positioned to promote screenings for other illnesses of which gay men are known to be at elevated risk for. If properly resourced, sexual health clinics could screen men for intimate partner violence, eating disorders, depression and/or offer smoking cessation programs to help reduce risk for heart disease and other serious health problems. While these are sensitive topics, gay men may be open to undergoing these additional assessments while receiving care within the trusted setting of a sexual health clinic. For example, the men in our study expressed an a priori level of comfort and expectation for discussing intimate/sexually explicit issues when going to sexual health clinics, and were psychologically prepared to talk about private and potentially anxiety-provoking issues in these settings. While the same is likely true for some men preparing to visit their primary care provider, such a pattern was far less prominent and not nearly as well articulated among our participants.

Finally, our research has potential implications for work with other communities or other health conditions. The findings presented here were derived from interviews with G/MSM, but this does not necessarily mean that the typologies and drivers are unique to gay men. For example, similar typologies, such as intentional care fragmentation, and similar drivers, such as stigma concerns, are likely to be seen in the mental healthcare field, where longstanding prejudicial attitudes about mental illness may lead some individuals to segregate psychiatric or psychological services from other forms of care [13], [14]. Ultimately, future research is needed to know if and how our behavioral typologies apply to other communities and other health conditions.

### Limitations

This study has several limitations. It was designed to explore the experiences of a specific set of individuals in a specific context, limiting the generalizability. The topics of sexual health and sexual healthcare that we explored during the in-depth interviews may be considered intimate, sensitive in nature, and highly personal, which could have influenced who specifically agreed to participate or what information they voluntarily disclosed. However, we will note that the participants who were in the study successfully articulated a variety of privacy-related considerations that influenced the specific care environments from which they received services (e.g., worries about how insurance companies or providers would respond, etc.). It seems likely that individuals more protective of their privacy (i.e., those less willing to participate given the sensitive nature of the study) would have identified similar types of concerns. Second, we did not include gay men who had never sought sexual health services, nor did we recruit men who have sex with men but do not identify as gay. Although many of the identified drivers of sexual healthcare decision-making would potentially be applicable to them, we cannot state with certainty that they would be the primary determinants of these men's healthcare choices. Obtaining their perspectives might provide additional insights into decision-making around sexual healthcare. Third, we collected data prior to the availability of pre-exposure prophylaxis and over-the-counter rapid HIV test kits. As such, our findings do not reflect any changes in perceptions or attitudes that may have emerged as a result of these two important advances in HIV prevention.

## Conclusions

With the implementation of the Patient Protection and Affordable Care Act in January 2014, public health researchers foresee opportunities to reduce health disparities and to reframe sexual health [72]. We are also optimistic and anticipate that gay and other MSM will be a part of the rising demand on the primary healthcare system. In particular, two important changes to the healthcare system may have a critical impact on G/MSM sexual healthcare. First, HIV and STI screening services are among the 15 preventative services that will be paid for by insurers [73]. This means that for those G/MSM for whom lack of insurance was a factor that drove them to seek screening outside of a PCP, this barrier will be effectively removed. And second, citizens will have access to insurance coverage regardless of their health status or pre-existing conditions [74]. Theoretically speaking, the new law will prevent insurance companies from discriminating against men presenting to their PCP for an HIV test. Thus, G/MSM concerned about being ‘blacklisted’ from insurers will benefit from learning about this structural and durable change in the law. We recommend that future research include evaluating the impact of the Affordable Care Act on health disparities associated with gay and other men who have sex with men as well as monitor whether men fragment or consolidate sexual healthcare and primary care. Whether fragmentation continues or not will be an indicator of whether sexual health services can be effectively delivered in primary care settings or whether specialized settings focused exclusively on sexual healthcare delivery are more acceptable and lead to improved health outcomes. We hypothesize that despite structural changes in the financing and delivery of healthcare services, G/MSM will continue to fragment sexual healthcare apart from primary care.

In counties with high levels of HIV prevalence, Departments of Public Health officials are considering where to locate new combination bio-behavioral HIV prevention interventions. Our findings can inform these decisions. Evidence presented here suggests that G/MSM tend to see sexual healthcare centers as the more logical location to receive sexual healthcare and importantly, they indicated a substantial level of skepticism and resistance to the notion that such services should be obtained in primary care settings. While the beliefs about primary care settings may not be accurate (i.e., insurance monitoring), they do drive men's behaviors and, thus, are an important consideration in the rollout of new clinic-based HIV prevention interventions. Since sexual health constitutes the majority of health needs for most young G/MSM, it seems intuitive to build their healthcare system around this need–yet; the current trend in healthcare delivery (centering care on a primary care medical home) seems to be pushing in the opposite direction. From a patient-centered perspective, we believe it would be preferable to adapt future healthcare models of care to match existing patient preferences rather than trying to push G/MSM to conform to a model that traditionally has not served them well. To that end, we recommend that policymakers and clinicians consider including more primary care services within sexual healthcare settings.

## References

[1] Gee R (2006) Primary care health issues among men who have sex with men. J Am Assoc Nurse Pract 18: 144–153. 10.1111/j.1745-7599.2006.00117.x 16573727

[2] Mayer KH, Bradford JB, Makadon HJ, Stoll R, Goldhammer H, et al (2008) Sexual and gender minority health: What we know and what needs to be done. Am J Public Health 98: 989–995. 18445789 10.2105/AJPH.2007.127811 PMC2377288

[3] Centers for Disease Control and Prevention (2012) HIV Surveillance Report, 2010 22: 1–79. Available: http://www.cdc.gov/hiv/topics/surveillance/resources/reports/. Accessed 2013 Mar 8.

[4] Purcell DW, Johnson CH, Lansky A, Prejean J, Stein R, et al. (2012) Estimating the Population Size of Men Who Have Sex with Men in the United States to Obtain HIV and Syphilis Rates. Open AIDS J 6: 98–107. Available: http://www.ncbi.nlm.nih.gov/pmc/articles/PMC3462414/. Accessed 2013 Mar 8. 10.2174/1874613601206010098 PMC3462414 23049658

[5] Centers for Disease Control and Prevention (2010) Viral Hepatitis: Gay and Bisexual Men's Health. Available: http://www.cdc.gov/msmhealth/viral-hepatitis.htm. Accessed 2013 Mar 8.

[6] Centers for Disease Control and Prevention (2008) Persons tested for HIV- United States, 2006. MMWR Morb Mortal Wkly Rep 57: 845–849. 18685551

[7] Wall KM, Khosropour CM, Sullivan PS (2010) Offering of HIV Screening to Men Who Have Sex With Men by Their Health Care Providers and Associated Factors. J Int Assoc Provid AIDS Care 9: 284–288. 10.1177/1545109710379051 PMC3637046 20841438

[8] Johnson CV, Mimiaga MJ, Reisner SL, Tetu AM, Cranston K, et al (2009) Health Care Access and Sexually Transmitted Infection Frequency Among At-Risk Massachusetts Men Who Have Sex With Men. Am J Public Health 99: 187–192. 10.2105/AJPH.2007.127464 PMC2724956 19218176

[9] Merson MH, O'Malley J, Serwadda D, Apisuk C (2008) The history and challenge of HIV prevention. Lancet 372: 475–488. 18687461 10.1016/S0140-6736(08)60884-3

[10] Hood JE, Friedman AL (2011) Unveiling the hidden epidemic: A review of stigma associated with sexually transmitted infections. Sex Health 8: 159–170. 21592429 10.1071/SH10070

[11] The Henry J. Kaiser Family Foundation (2013) Fact Sheet: The Ryan White Program. Available: http://www.kff.org/hivaids/7582.cfm. Accessed 2013 Mar 8.

[12] Saag MS (2009) Ryan White: An unintentional home builder. AIDS Read 19: 166–168. 19554735

[13] Parcesepe AM, Cabassa LJ (2012) Public stigma of mental illness in the United States: A systematic literature review. Adm Policy Ment Health. Available: http://link.springer.com/article/10.1007%2Fs10488-012-0430-z . Accessed 2013 Mar 8. 10.1007/s10488-012-0430-z PMC3835659 22833051

[14] Roll JM, Kennedy J, Tran M, Howell D (2013) Disparities in unmet need for mental health services in the United States, 1997–2010. Psychiatr Serv 64: 80–82. 23280460 10.1176/appi.ps.201200071

[15] Lauby JL, Milnamow M (2009) Where G/MSM have their first HIV test: Differences by race, income, and sexual identity. Am J Mens Health 3: 50–59. 19477719 10.1177/1557988307313465

[16] Mimiaga MJ, Goldhammer H, Belanoff C, Tetu AM, Mayer KH (2007) Men who have sex with men: perceptions about sexual risk, HIV and sexually transmitted disease testing, and provider communication. Sex Transm Dis. 34: 113–119. 10.1097/01.olq.0000225327.13214.bf 16810121

[17] UNAIDS. Available: www.unaids.org. Accessed 2013 Mar 8.

[18] Vermund S, Hayes R (2013) Combination Prevention: New Hope for Stopping the Epidemic. Curr HIV/AIDS Rep 10. Available: http://link.springer.com/content/pdf/10.1007%2Fs11904-013-0155-y. Accessed 2013 Mar 8. 10.1007/s11904-013-0155-y PMC3642362 23456730

[19] The HIV Modelling Consortium Treatment as Prevention Editorial Writing Group (2012) HIV treatment as prevention: models, data, and questions–towards evidence-based decision-making. PLoS Med 9: e1001259 Available: http://www.plosmedicine.org/article/info%3Adoi%2F10.1371%2Fjournal.pmed.100125. Accessed 2013 Mar 8. 10.1371/journal.pmed.1001259 PMC3393655 22802739

[20] Wilson D, Halperin D (2008) Know your epidemic, know your response: a useful approach, if we get it right. Lancet 372: 423–426. 18687462 10.1016/S0140-6736(08)60883-1

[21] Kurth AE, Celum C, Baeten JM, Vermund SH (2011) Wasserheit JN (2011) Combination HIV Prevention: Significance, Challenges, and Opportunities. Curr HIV/AIDS Rep 8: 62–72. 20941553 10.1007/s11904-010-0063-3 PMC3036787

[22] Padian NS, McCoy SI, Karim SS, Hasen N, Kim J, et al (2011) HIV prevention transformed: the new prevention research agenda. Lancet 378: 269–78. 21763938 10.1016/S0140-6736(11)60877-5 PMC3606928

[23] Mayer KH, Venkatesh KK (2010) Antiretroviral therapy as HIV prevention: Status and prospects. Am J Public Health 100: 1867–1876. 20724682 10.2105/AJPH.2009.184796 PMC2936983

[24] Cohen MS, Chen YQ, McCauley M, Gamble T, Hosseinipour MC, et al (2011) Prevention of HIV-1 infection with early antiretroviral therapy. N Engl J Med 365: 493–505. 21767103 10.1056/NEJMoa1105243 PMC3200068

[25] Quinn TC, Wawer MJ, Sewankambo N, Serwadda D, Li C, et al (2000) Viral load and heterosexual transmission of human immunodeficiency virus type 1. Rakai Project Study Group. N Engl J Med 342: 921–929. 10738050 10.1056/NEJM200003303421303

[26] Chang LW, Serwadda D, Quinn TC, Wawer MJ, Gray RH, et al (2013) Combination implementation for HIV prevention: Moving from clinical trial evidence to population-level effects. Lancet Infect Dis 13: 65–76. 23257232 10.1016/S1473-3099(12)70273-6 PMC3792852

[27] Centers for Disease Control and Prevention (2011) Vital signs: HIV prevention through care and treatment–United States. MMWR Morb Mortal Wkly Rep 60: 1618–1623. 22129997

[28] Weiser SD, Wolfe WR, Bangsberg DR (2004) The HIV Epidemic Among Individuals with Mental Illness in the United States. Curr Infect Dis Rep 6: 404–410. 15461893 10.1007/s11908-004-0041-2

[29] Hu YW, Kinsler JJ, Sheng Z, Kang T, Bingham T, et al (2012) Using laboratory surveillance data to estimate engagement in care among persons living with HIV in Los Angeles County, 2009. AIDS Patient Care STDS 26: 471–478. 22731500 10.1089/apc.2011.0371

[30] Ulett KB, Willig JH, Lin HY, Routman JS, Abroms S, et al (2009) The therapeutic implications of timely linkage and early retention in care. AIDS Patient Care STDS 23: 41–49. 19055408 10.1089/apc.2008.0132 PMC2733237

[31] Riley ED, Neilands TB, Moore K, Cohen J, Bangsberg DR, et al. (2012) Social, structural and behavioral determinants of overall health status in a cohort of homeless and unstably house HIV-infected men. PLoS One 7: e35207. Available: http://www.plosone.org/article/info%3Adoi%2F10.1371%2Fjournal.pone.0035207. Accessed 2013 Mar 8. 10.1371/journal.pone.0035207 PMC3338834 22558128

[32] Karim QA, Karim SS, Frohlich JA, Grobler AC, Baxter C, et al (2010) Effectiveness and safety of tenofovir gel, an antiretroviral microbicide, for the prevention of HIV infection in women. Science 329: 1168–1174. 20643915 10.1126/science.1193748 PMC3001187

[33] Grant RM, Lama JR, Anderson PL, McMahan V, Liu AY, et al (2010) Pre-exposure chemoprophylaxis for HIV prevention in men who have sex with men. N Engl J Med 363: 2587–2599. 21091279 10.1056/NEJMoa1011205 PMC3079639

[34] Buchbinder SP, Liu A (2011) Pre-exposure prophylaxis and the promise of combination prevention approaches. AIDS Behav 15: 72–79. 10.1007/s10461-011-9894-1 PMC3064892 21331801

[35] Underhill K, Operario D, Mimiaga MJ, Skeer MR, Mayer KH (2010) Implementation science of pre-exposure prophylaxis: Preparing for public use. Curr HIV/AIDS Rep 7: 210–219. 20820971 10.1007/s11904-010-0062-4 PMC3012127

[36] Underhill K, Operario D, Skeer M, Mimiaga M, Mayer K (2010) Packaging PrEP to prevent HIV: An integrated framework to plan for pre-exposure prophylaxis implementation in clinical practice. J Acquir Immune Defic Syndr 55: 8–13. 21423876 10.1097/qai.0b013e3181e8efe4 PMC3058525

[37] Moustakas C (1994) Phenomenological Research Methods. London: Sage Publications, Inc. 208 p.

[38] Ritchie J, Spencer L (1994) Qualitative data analysis for applied policy research. In: Bryman A, Burgess RG, editors. Analyzing Quantitative Data. London: Routledge. 173–194.

[39] Muhr T (1997) ATLAS.ti 5: The Knowledge Workbench. Berlin: Scientific Software Development.

[40] Creswell JW (1995) Research Design: Qualitative and Quantitative Approaches. Thousand Oaks: Sage Publications, Inc. 248 p.

[41] Tong A, Sainsbury P, Craig J (2007) Consolidated criteria for reporting qualitative research (COREQ): a 32-item checklist for interviews and focus groups. Int J Qual Health Care 19: 349–357. 17872937 10.1093/intqhc/mzm042

[42] Goffman E (1963) Stigma: Notes on the Management of Spoiled Identity. New York: Simon and Schuster, Inc. 168 p.

[43] Aberg JA, Kaplan JE, Libman H, Emmanuel P, Anderson JR, et al (2009) Primary Care Guidelines for the Management of Persons Infected with Human Immunodeficiency Virus: 2009 Update by the HIV Medicine Association of the Infectious Diseases Society of America. Clin Infect Dis 49: 651–681. 19640227 10.1086/605292

[44] Wolitski R, Fenton K (2011) Sexual Health, HIV and Sexually Transmitted Infections among Gay, Bisexual, and Other Men Who Have Sex with Men in the United States. AIDS Behav 15: 9–17. 21331797 10.1007/s10461-011-9901-6

[45] Ostrow DG, Stall R (2008) Alcohol, tobacco, and drug use among gay and bisexual men. In Wolitski RJ, Stall R, Valdiserri RO, editors. Unequal opportunity: Health disparities affecting gay and bisexual men in the United States. New York: Oxford University Press. 121–158.

[46] Centers for Disease Control and Prevention (2010) Substance Abuse: Gay and Bisexual Men's Health. Available: http://www.cdc.gov/msmhealth/substance-abuse.htm. Accessed 2013 Mar 8.

[47] Irwin TW, Morgenstern H, Parsons JT, Wainberg M, Labouvie E (2006) Alcohol and sexual HIV risk behavior among problem drinking men who have sex with men: An event level analysis of timeline follow-back data. AIDS Behav 10: 299–307. 16482407 10.1007/s10461-005-9045-7

[48] Wong CF, Kipke MD, Weiss G (2008) Risk factors for alcohol use, frequent use, and binge drinking among young men who have sex with men. Addict Behav 33: 1012–1020. 18495364 10.1016/j.addbeh.2008.03.008 PMC2483958

[49] Stall R, Paul JP, Greenwood G, Pollack LM, Bein E, et al (2001) Alcohol use, drug use and alcohol-related problems among men who have sex with men: The Urban Men's Health Study. Addiction 96: 589–601. 11784456 10.1046/j.1360-0443.2001.961115896.x

[50] Padilla Y, Crisp C, Rew D (2010) Parental acceptance and illegal drug use among gay, lesbian, and bisexual adolescents: Results from a national survey. Soc Work 55: 265–275. 20632661 10.1093/sw/55.3.265

[51] Halkitis PN, Mukherjee PP, Palamar JJ (2009) Longitudinal modeling of methamphetamine use and sexual risk behaviors in gay and bisexual men. AIDS Behav 13: 783–791. 18661225 10.1007/s10461-008-9432-y PMC4669892

[52] World Heart Federation (2012) Cardiovascular disease risk factors. Available: http://www.world-heart-federation.org/cardiovascular-health/cardiovascular-disease-risk-factors/. Accessed 2013 Mar 8.

[53] Lee JG, Griffin GK, Melvin CL (2009) Tobacco use among sexual minorities in the USA, 1987 to May 2007: A systematic review. Tob Control 18: 275–282. Available: http://tobaccocontrol.bmj.com/content/18/4/275.long. Accessed 2013 Mar 8. 10.1136/tc.2008.028241 19208668

[54] Gruskin EP, Greenwood GL, Matevia M, Pollack LM, Bye LL (2007) Disparities in smoking between the lesbian, gay, and bisexual population and the general population in California. Am J Public Health 97: 1496–1502. 17600265 10.2105/AJPH.2006.090258 PMC1931451

[55] Greenwood GL, Paul JP, Pollack LM, Binson D, Catania JA, et al. (2005) Tobacco use and cessation among a household-based sample of U.S. urban men who have sex with men. Am J Public Health 95: 145–151. Available: http://www.ncbi.nlm.nih.gov/pmc/articles/PMC1449276/pdf/0950929.pdf. Accessed 2013 Mar 8. 10.2105/AJPH.2003.021451 PMC1449867 15623875

[56] Asencio M, Blank T, Descartes L (2009) The prospect of prostate cancer: A challenge for gay men's sexualities as they age. Sex Res Social Policy 6: 38–51.

[57] Bowen DJ, Boehmer U (2007) The lack of cancer surveillance data on sexual minorities and strategies for change. Cancer Causes Control 18: 343–349. 17325829 10.1007/s10552-007-0115-1

[58] Heslin KC, Gore JL, King WD, Fox S (2008) Sexual orientation and testing for prostate and colorectal cancers among men in California. Med Care 46: 1240–1248. 19300314 10.1097/MLR.0b013e31817d697f PMC2659454

[59] Chin-Hong PV, Vittinghoff E, Cranston RS, Browne L, Buchbinder S, et al (2005) Age-related prevalence of anal cancer precursors in homosexual men: The EXPLORE study. J Natl Cancer Inst 97: 896–905. 15956651 10.1093/jnci/dji163

[60] McRee AL, Reiter PL, Chantala K, Brewer NT (2010) Does framing human papillomavirus vaccine as preventing cancer in men increase vaccine acceptability? Cancer Epidemiol Biomarkers Prev 19: 1937. 20647398 10.1158/1055-9965.EPI-09-1287 PMC2919615

[61] Herek GM (2009) Hate crimes and stigma-related experiences among sexual minority adults in the United States: Prevalence estimates from a national probability sample. J Interpers Violence 24: 54–74. 18391058 10.1177/0886260508316477

[62] Willis DG (2004) Hate crimes against gay males: An overview. Issues Ment Health Nurs 25: 115–132. 14726266 10.1080/01612840490268090

[63] Houston E, McKirman DJ (2007) Intimate partner abuse among gay and bisexual men: Risk correlates and health outcomes. J Urban Health 84: 681–690. 17610158 10.1007/s11524-007-9188-0 PMC2231846

[64] Siconolfi D, Halkitis PN, Allomong TW (2009) Body dissatisfaction and eating disorders in a sample of gay and bisexual men. Int J Mens Health 8: 254–264.

[65] Donald R, McCreary TB, Hildebrandt LJ, Heinberg MB, Thompson JK (2007) A review of body image influences on men's fitness goals and supplement use. Am J Mens Health 1: 307–316. 19482812 10.1177/1557988306309408

[66] Deputy NP, Boehmer U (2010) Determinants of body weight among men of different sexual orientation. Prev Med 51: 129–131. 20510272 10.1016/j.ypmed.2010.05.010

[67] Cochran SD, Mays VM, Alegria M, Ortega AN, Takeuchi D (2007) Mental health and substance use disorders among Latino and Asian American lesbian, gay, and bisexual adults. J Consult Clin Psychol 75: 785–794. 17907860 10.1037/0022-006X.75.5.785 PMC2676845

[68] Gilman SE, Cochran SD, Mays VM, Hughes M, Ostrow D, et al (2001) Risk of psychiatric disorders among individuals reporting same-sex sexual partners in the National Comorbidity Survey. Am J Public Health 91: 933–939. 11392937 10.2105/ajph.91.6.933 PMC1446471

[69] Berg MB, Mimiaga MJ, Safren SA (2008) Mental health concerns of gay and bisexual men seeking mental health services. J Homosex 54: 293–306. 18825866 10.1080/00918360801982215

[70] Burgess D, Tran A, Lee R, Van Ryn M (2008) Effects of perceived discrimination on mental health and mental health services utilization among gay, lesbian, bisexual and transgender persons. J LGBT Health Res 4: 43. 10.1080/15574090802226626 19042907

[71] Bostwick WB, Boyd CJ, Hughes TL, MCabe SE (2009) Dimensions of sexual orientation and the prevalence of mood and anxiety disorders in the United States. Am J Public Health 100: 468–475. Available: http://www.ncbi.nlm.nih.gov/pmc/articles/PMC2820045/?tool=pubmed. Accessed 2013 Mar 8. 10.2105/AJPH.2008.152942 PMC2820045 19696380

[72] Lanier Y, Madeline SY (2013) Reframing the Context of Preventive Health Care Services and Prevention of HIV and Other Sexually Transmitted Infections for Young Men: New Opportunities to Reduce Racial/Ethnic Sexual Health Disparities. Am J Public Health 103: 262–269. 23237172 10.2105/AJPH.2012.300921 PMC3558761

[73] U.S. Department of Health & Human Services (2010) Preventive Services Covered Under the Affordable Care Act. Available: http://www.healthcare.gov/news/factsheets/2010/07/preventive-services-list.html. Accessed 2013 Mar 8.

[74] U.S. Department of Health & Human Services (2010) Pre-Existing Condition Insurance Plan (PCHIP). Available: http://www.healthcare.gov/law/features/choices/pre-existing-condition-insurance-plan/index.html. Accessed 2013 Mar 8.

